# Evaluation of cinitapride's efficacy and safety in treating functional dyspepsia with overlapping symptoms: a real-world study in Chinese healthcare settings

**DOI:** 10.1590/1806-9282.20241628

**Published:** 2025-05-02

**Authors:** Bing Yan, Shimin Wang, Jian Chen, Xiaolin Zhong, Bangmao Wang, Yumei Wu, Ji Wu, Nina Zhang, Jing Sun, Duowu Zou

**Affiliations:** 1 Chongqing Medical University, The Second Affiliated Hospital, Department of Gastroenterology – Chongqing, China.; 2 Shanghai Jiao Tong University, School of Medicine, Ruijin Hospital, Department of Gastroenterology – Shanghai, China.; 3 Zhaoqing First People's Hospital, Department of Gastroenterology – Zhaoqing, China.; 4 Affiliated Hospital of Southwest Medical University, Department of Gastroenterology – Luzhou, China.; 5 Tianjin Medical University General Hospital, Department of Gastroenterology – Tianjin, China.; 6 The Third People's Hospital of Shenzhen, Department of Gastroenterology – Shenzhen, China.; 7 Foshan Fosun Chancheng Hospital, Department of Gastroenterology – Foshan, China.; 8 Nanjing Drum Tower Hospital (The Affiliated Hospital of Nanjing University Medical School), Department of Gastroenterology – Nanjing, China.

**Keywords:** Real-world, Cinitapride, Effectiveness, Safety, Dyspepsia, Comorbidity

## Abstract

**OBJECTIVE:**

Cinitapride, a gastrointestinal prokinetic, is commonly used for treating functional dyspepsia. However, large-scale, real-world data on its efficacy, especially in patients with overlapping symptoms, are limited. The aim of this study was to evaluate the clinical effectiveness and safety of cinitapride in Chinese patients with functional dyspepsia, including those with overlapping symptoms, in a real-world setting.

**METHODS:**

In this single-arm, prospective, multicentric study, 1,012 Chinese outpatients with functional dyspepsia and functional dyspepsia overlapping with gastroesophageal reflux disease, irritable bowel syndrome, and/or functional constipation were treated with cinitapride (1 mg t.i.d.) from May 2019 to March 2021. Symptom improvement was assessed at weeks 2 and 4, with adverse events recorded.

**RESULTS:**

At weeks 2 and 4, the overall symptom improvement rate was 62.4 and 90.9%, respectively. Subgroup improvement rates were as follows: functional dyspepsia-gastroesophageal reflux disease, 86.8%; functional dyspepsia-irritable bowel syndrome, 96.2%; functional dyspepsia-functional constipation, 91.7%; and functional dyspepsia-gastroesophageal reflux disease-irritable bowel syndrome, 67.6%. functional dyspepsia patients showed statistically significantly higher improvement than those with overlapping symptoms at weeks 2 (p=0.018) and 4 (p=0.009). The dyspepsia symptom score decreased by 51.0% at week 2 and 74.4% at week 4 (p<0.001). The most common adverse event was asymptomatic electrocardiogram abnormalities (n=8).

**CONCLUSION:**

Cinitapride is effective and well-tolerated in treating functional dyspepsia and functional dyspepsia-overlapping gastroesophageal reflux disease, irritable bowel syndrome, and functional constipation in Chinese patients.

## INTRODUCTION

Functional dyspepsia (FD), a common gastrointestinal disorder affecting around 20% of the population, is characterized by recurrent epigastric pain, postprandial fullness, and early satiety. Approximately 80% of patients with FD show no structural or biochemical abnormalities. The pathogenesis of FD is thought to involve disordered brain–gut signaling, leading to issues, such as disturbed gastroduodenal motility, visceral hypersensitivity, and altered gut microbiota and immune responses^1^. FD frequently overlaps with other gastrointestinal disorders, including gastroesophageal reflux disease (GERD), irritable bowel syndrome (IBS), and/or functional constipation (FC). Studies report a 7.41% GERD-FD overlap, with 41.15% of GERD patients experiencing FD symptoms, and a 64% FD-IBS overlap in functional gastrointestinal disorder (FGID) patients^2,3^. Overlapping FD generally results in more severe symptoms, higher depression rates, impaired quality of life, and a greater economic and societal burden.

Prokinetics are a key component of FD pharmacotherapy and are recommended for symptom control in clinical guidelines^4^. Traditional effective gastrointestinal tract promotility agents such as domperidone and cisapride were restricted for usage or withdrawn due to life-threatening cardiac arrhythmias. Considering the prevalence of FD and FD with overlapping symptoms worldwide, novel alternative gastrointestinal prokinetic drugs with good safety and efficacy are urgently needed.

Cinitapride, a benzamide-derived molecule, which exhibits agonistic activity at 5-hydroxytryptamine1 (HT1) and 5-hydroxytryptamine4 (5HT4) receptors and antagonistic activity at 5HT2 and D2 dopaminergic receptors^5^, has been widely used in mild-to-moderate FD and GERD^6-8^. It has been proved that it is also effective for IBS and FC symptoms^9,10^. Cinitapride is of great benefit in accelerating gastrointestinal motility among preclinical studies and phase III and phase IV clinical trials^11-13^. Nevertheless, there is a lack of real-world data evaluating its effect on FD-overlapping symptoms.

Therefore, we investigated the clinical effectiveness and tolerability of cinitapride in Chinese patients with FD alone and FD with overlapping symptoms in a real-world setting, thus providing evidence for the treatment.

## METHODS

### Patients

From May 2019 to March 2021, 1,012 consecutive gastroenterology outpatients with FD were recruited across 12 sites in China. All patients met Rome IV diagnostic criteria, having experienced postprandial fullness, early satiety, or mid-upper abdominal discomfort for at least 6 months. Exclusion criteria included structural gastrointestinal diseases, mental impairments, allergies to cinitapride, or conditions likely to result in a follow-up loss.

Written informed consent was obtained from all patients prior to study participation. This study adhered to Good Clinical Practice (GCP) guidelines and the Declaration of Helsinki. Ethical approval was obtained from the Ruijin Hospital's Ethics Committee and all engaged centers. The study was registered with ChiCTR (www.chictr.org.cn, ChiCTR2000030949).

### Study design

This was a multicentric, prospective, observational study conducted in a real-world setting. A face-to-face questionnaire survey at baseline was conducted for eligible patients. The questionnaire was designed based on Rome IV criteria, which mainly included patient's general information, relevant gastroscopy, barium meal (gastric emptying) test results, FD-related symptoms, and quality of life assessment. All enrolled patients were instructed to take cinitapride (1 mg, t.i.d.) for 4 weeks. Follow-ups were performed through on-site questionnaires or telephone questionnaires at the second and fourth weeks of the treatment.

### Effectiveness evaluation

A questionnaire was used to assess dyspeptic symptoms, including postprandial fullness, early satiety, mid-upper abdominal pain, and burning discomfort. Symptom severity was self-assessed on a 5-point Likert scale: none (0), mild (1), moderate (2), severe (3), and extreme (4). Symptom frequency was also rated on a 5-point scale: none (0), <1 day/week (1), 1 day/week (2), >1 day/week (3), and every day (4)^14^. The total symptom score was calculated by multiplying each symptom's severity by its frequency, with a maximum score of 64. Treatment was deemed effective if the symptom improvement rate exceeded 50%. Primary endpoints were improvement rates at weeks 2 and 4.

### Safety assessment

The occurrence of adverse events (AEs) was evaluated and recorded during the whole study. AE severity was categorized into mild, moderate, or severe, and the extent of severity refers that it led to hospitalization or death. Patients recruited in Shanghai Ruijin Hospital and Qingyuan People's Hospital were selected to take laboratory examinations including surface electrocardiogram (ECG), liver function tests (alanine aminotransferase [ALT] and aspartate aminotransferase [AST]), and renal function (creatinine) examinations at baseline and at week 4, thus actively monitoring the safety of the heart, liver, and kidney.

### Statistical analysis

Statistical analysis was performed using SAS version 9.4 (SAS Institute Inc.) on full analysis set (FAS) and safety analysis set (SS). Paired t-test or Wilcoxon signed-rank test was used for within-group comparisons and t-test or analysis of variance for between-group comparisons. Chi-square or Fisher's exact test was applied to categorical data. Statistical significance was set at p<0.05 with a 95% confidence interval.

## RESULTS

### Basline characteristics

In total, 1,012 patients receiving cinitapride treatment and at least one post-treatment safety evaluation were included in the SS, while 983 patients with post-baseline efficacy data were included in the FAS. Of the FAS, 68.9% (677/983) had FD alone and 31.1% (306/983) had overlapping symptoms, including FD-GERD (49.7%), FD-IBS (35.3%), FD-FC (3.6%), FD-GERD-IBS (11.1%), and FD-IBS-FC (0.3%). A majority of the patients were aged 31–50 years (50.6%), with a balanced gender distribution (51.8% of females). The body mass index of 74.3% was within the normal range. Disease duration varied, with 50.7% having FD for 1–12 months and 38.1% for over 1 year. Most patients (66.9%) were treated with cinitapride monotherapy, while others received additional prokinetics, gastric mucosal protectors, antacids, or anti-*Helicobacter pylori* (Hp) drugs (Table 1).

**Table 1:** Demographic information and baseline characteristics of the enrolled patients.

	Patients (n=983) (n [%])		Patients (n=983) (n [%])
Age, years		Hp (+)	99 (10.1)
18–30	109 (11.1)	drugs	
31–50	497 (50.6)	Cinitapride alone	658 (66.9)
51–60	212 (21.6)	Prokinetics	196 (19.9)
>60	163 (16.6)	Mucosal protective drugs	112 (11.4)
Gender (female)	509 (51.8)	Antacids	178 (18.1)
BMI (18.5–23.9, kg/m^2^)	730 (74.3)	Anti-Hp treatment	41 (4.2)
Course of disease		Diagnosis	
New diagnosis	9 (0.9)	FD	677 (68.9)
1 week–1 month	85 (8.6)	FD-overlapping symptoms	306 (31.1)
1 month–1 year	498 (50.7)	FD-GERD	152 (49.7)
>1 year	375 (38.1)	FD-IBS	108 (35.3)
Never smoke	696 (70.8)	FD-FC	11 (3.6)
Never drink	517 (52.6)	FD-GERD-IBS	34 (11.1)
Had Hp check	72 (7.3)	FD-IBS-FC	1 (0.3)

BMI: body mass index; Hp: *Helicobacter pylori*; FD: functional dyspepsia; FD-GERD: functional dyspepsia-gastroesophageal reflux disease; FD-IBS: functional dyspepsia-irritable bowel syndrome; FD-FC: functional dyspepsia-functional constipation; FD-GERD-IBS: functional dyspepsia-gastroesophageal reflux disease-irritable bowel syndrome; FD-IBS-FC: functional dyspepsia-irritable bowel syndrome-functional constipation.

### Primary endpoint: effective rate of total symptom score improvement

Till March 2021, 983 patients with FD completed 4 weeks of treatment with cinitapride. As shown in Table 2, after 2 and 4 weeks of cinitapride treatment, the effective rate of total symptom score improvement of the total FD population had reached 62.4 and 90.9%, respectively. After 2 weeks of cinitapride treatment, the effective rate in the FD-alone group and FD combined with overlapping symptoms group was 65.8 and 55.0%, respectively. While after 4 weeks, the effective rate of these two groups had reached 92.2 and 88.1%, respectively. The FD group exhibited statistically significantly higher improvement rates in total symptom score compared to the FD with overlapping symptoms group, with a 9.2% difference at 2 weeks (p=0.018) and a 4.1% difference at 4 weeks (p=0.009).

**Table 2:** Effective rate of total symptom score improvement among overall and subgroups.

	2 weeks	p-value	4 weeks	p-value
Overall samples	62.4% (598/958)		90.9% (871/958)	
FD alone	65.8% (434/660)	0.018*	92.2% (604/655)	0.009*
FD-overlapping symptoms	55.0% (164/298)		88.1% (267/303)	
FD-GERD	56.1% (83/148)	0.422#	86.8% (131/151)	0.001#
FD-IBS	58.7% (61/104)		96.2% (102/106)	
FD-GERD-IBS	35.3% (12/34)		67.6% (23/34)	
FD-FC and FD-IBS-FC	66.7% (8/12)		91.7% (11/12)	

* FD-alone group compared with the FD-overlapping symptom group;

# comparisons between FD-overlapping symptom subgroups.

p-value was obtained by Pearson's chi-square test. FD: functional dyspepsia; FD-GERD: functional dyspepsia-gastroesophageal reflux disease; FD-IBS: functional dyspepsia-irritable bowel syndrome; FD-GERD-IBS: functional dyspepsia-gastroesophageal reflux disease-irritable bowel syndrome; FD-FC: functional dyspepsia-functional constipation; FD-IBS-FC: functional dyspepsia-irritable bowel syndrome-functional constipation.

Improvement rates in the total symptom score were not statistically significantly different between the subgroups of FD-overlapping symptoms at week 2 (p=0.422). However, with prolonged drug administration until 4 weeks, the effective rate of subgroups was statistically significantly different (p=0.001), while it was 86.8, 96.2, 67.6, and 91.7% in the FD-GERD, FD-IBS, FD-GERD-IBS, and FD-FC and FD-IBS-FC subgroups, respectively.

### Secondary endpoints: dyspepsia symptom improvement rate

After 2 and 4 weeks of cinitapride treatment, the average dyspepsia symptom score in the total population decreased by 51.0 and 74.4%, respectively. At 4 weeks, all four main symptoms of dyspepsia improved by over 70%, with early satiety showing the highest improvement (80.6%). Improvement rates at 4 weeks were statistically significantly higher compared to 2 weeks (p<0.001). Patients with FD alone showed higher symptom improvement rates than those with overlapping symptoms, with total score differences at 2 weeks (53.77 vs. 46.68%, p=0.001) and 4 weeks (77.15 vs. 69.34%, p<0.001). Early satiety improved the most in the FD-IBS group, with an 87.2% decrease (p=0.002), while postprandial fullness showed the greatest improvement in the FD-GERD group (71.8%, p=0.073). Epigastric pain/burning improved significantly in the FD-IBS group, with symptom relief rates of 83.8 and 92.8% (p=0.001 and p=0.004, respectively). Additionally, the FD-IBS group had the largest decrease in the total symptom score, showing a reduction of 73.18% (p=0.002).

### Safety analysis

All patients who received cinitapride therapy and underwent at least one post-treatment safety evaluation were enrolled in the SS. Of the 1,012 patients, 3 cases of AE were observed during follow-up at week 2, including gastric distention (n=1), dizziness (n=1), and nausea (n=1), with an incidence rate of 0.29%, of which no cases of serious adverse events (SAEs) were reported. All these symptoms were mild and recovered soon after drug discontinuation.

Considering the safety of cinitapride on the heart, liver, and kidney, patients at Ruijin Hospital and Qingyuan People's Hospital (n=94) were actively monitored by laboratory examination at baseline and 4 weeks after initial treatment. In these 94 samples, 8 people experienced mild ECG abnormalities including alteration in ST-T waves (n=4), sinus arrhythmia (n=3), and high left ventricular voltage (n=1), while no cases of prolonged QT interval were found. One patient showed slight elevations in AST (42 IU/L, normal level 8–40 IU/L) (Table 3). Cinitapride treatment resulted in an 8.1% incidence of AEs after 4 weeks.

**Table 3:** Adverse events and laboratory abnormalities during treatment.

AEs			2 weeks (n [%])	4 weeks (n [%])
Any AE			3 (0.29)	9 (8.1)
Serious adverse reactions			0	0
	Gastric distention		1	0
	Dizziness		1	0
	Nausea		1	0
Abnormal laboratory examination				
	ECG abnormalities			8 (7.2)
		Alteration in ST-T waves		4
		High left ventricular voltage		1
		Sinus arrhythmia (tachycardia/bradycardia)		3
		Prolonged QT interval		0
Elevations in ALT				0
Elevations in AST				1 (0.9)
Elevated serum creatinine				0

ECG: electrocardiogram; ALT: alanine aminotransferase; AST: aspartate aminotransferase; AE: adverse event; ST-T: ST segment and T wave abnormalities; QT: corrected QT interval (Bazett formula).

## DISCUSSION

Cinitapride, a benzamide derivative, stimulates 5-HT4 and 5-HT1 and blocks the 5-HT2 and D2 receptors. The gastrointestinal motility effect of cinitapride is mainly achieved by enhancing acetylcholine release via stimulating 5-HT4 receptors and antagonizing the dopamine D2 receptor. Cinitapride demonstrates greater in vitro stimulatory activity on guinea pig intestinal smooth muscle than metoclopramide, suggesting a mechanism involving enhanced acetylcholine release from intramural cholinergic neurons. Previous studies have also demonstrated that cinitapride has gastroprotective effects to improve gastric ulceration and secretion in rats, which could be partly explained through 5-HT-dependent mechanisms via 5-HT_2_R antagonism and 5-HT_1_R agonism.

Although results on the effectiveness of cinitapride have been inconsistent, clinical guidelines recommend prokinetic agents as first-line treatment for FD^15^. Several randomized controlled trials have investigated cinitapride's efficacy in FD patients. Phase I studies showed similar therapeutic efficacy between metoclopramide and cinitapride, with good tolerability in healthy Chinese subjects at doses of 1–4 mg. Phase II studies demonstrated better efficacy and tolerability of cinitapride compared to metoclopramide and placebo for gastrointestinal transit disorders. A Phase III trial confirmed cinitapride's non-inferiority to domperidone in patients with mild-to-moderate postprandial distress syndrome-predominant FD. Additionally, a Phase IV study in Pakistan showed that cinitapride effectively controlled dyspepsia symptoms and improved patients’ quality of life. However, real-world data on cinitapride's effectiveness, especially in patients with overlapping symptoms, remain limited. This study is the first real-world evaluation of cinitapride's efficacy and safety in FD and FD with overlapping symptoms.

In the current study, cinitapride proved effective in treating both FD and FD with overlapping symptoms. Following 2 and 4 weeks of treatment, the effective rate of total symptom score improvement reached 62.4 and 90.9%, respectively. Dyspepsia symptom scores decreased by 51.0% at week 2 and 74.4% at week 4. Significant improvements were seen in the four main dyspeptic symptoms—postprandial fullness, early satiety, epigastric pain, and epigastric burning—at both time points, with the improvement rate for early satiety reaching 80.6% by week 4. A statistically significant improvement in dyspepsia symptom scores was observed between weeks 2 and 4 (p<0.001), highlighting the benefits of continued cinitapride use for at least 4 weeks.

In real-world settings, FD is often complicated by overlapping symptoms, which can worsen patient outcomes and reduce quality of life. This study, the first of its kind, evaluated cinitapride's efficacy in patients with overlapping symptoms. After 4 weeks, the FD-IBS subgroup showed the highest effective rate of total symptom score improvement (96.2%), while the FD-GERD, FD-GERD-IBS, and FD-FC and FD-IBS-FC subgroups had effective rates of 86.8, 67.6, and 91.7%, respectively. In the FD-IBS subgroup, early satiety showed the largest symptom score reduction, decreasing by 87.2% at week 4. The FD-GERD subgroup showed the most improvement in postprandial fullness (71.8%), while the FD-IBS subgroup demonstrated the most improvement in epigastric pain and burning, with symptom reductions of 83.8 and 92.8%, respectively. The FD-IBS subgroup also had the largest decrease in the total symptom score (73.2%). These results suggest that cinitapride's effectiveness varies depending on the specific overlapping symptoms, with the best outcomes seen in FD patients who also have IBS. Epidemiological studies have shown high rates of overlap between FD, GERD, and IBS, indicating potential shared pathophysiological mechanisms. This study's findings further support the theory that cinitapride may target common mechanisms underlying these overlapping conditions.

When comparing the efficacy of cinitapride in patients with FD alone versus those with overlapping symptoms, the results indicated that patients with FD alone responded better, with higher rates of total symptom score improvement and dyspepsia symptom improvement. The reduced efficacy in patients with overlapping symptoms may be due to the increased complexity and severity of their conditions. The precise mechanism by which cinitapride affects FD with overlapping symptoms is still unclear but may involve shared pathophysiological mechanisms, such as delayed gastric emptying, impaired gastric accommodation, and possibly the peripheral opioid system.

Adverse reactions to cinitapride, such as exanthema, sore throat, extrapyramidal symptoms, and unexplained increases in globulin levels, have been reported in previous studies^12,13,16^. However, no severe adverse events were noted in this study. Traditional prokinetic agents such as domperidone, mosapride, and cisapride were withdrawn due to cardiac safety concerns^17-19^, making cinitapride's safety profile particularly important. In this real-world study, cinitapride was well tolerated, with no signs of hepatorenal or cardiac toxicity. Mild ECG abnormalities were reported in eight cases, but none were symptomatic, and no QT interval prolongation was observed, consistent with prior studies^20^. A mild, asymptomatic increase in AST was noted in one patient, but no serious adverse reactions occurred throughout the study. Cinitapride is rapidly absorbed and primarily metabolized by CYP3A4 and CYP2C8, which may reduce the risk of drug interactions.

This is the first study to analyze the efficacy and safety of cinitapride on FD with overlapping symptoms and also the first study to be conducted in a real-world setting. This real-world, multicentric, prospective, non-interventional study with a large sample size is relatively persuasive. However, patient compliance management is usually regarded as a difficult part in real-world studies. Of the 1012 enrolled cases, 29 were excluded due to missing follow-up data, which might be due to that, after the treatment, a satisfactory effect has been achieved and the patient's willingness to return to hospital has decreased. Patient compliance is an important factor affecting the outcome of clinical studies, especially in real-world settings. In our study, we have taken a number of measures to improve patient compliance, such as patient education, medication guidance, and regular follow-ups. In future studies, incentives could be developed to encourage patients to follow up on time, such as providing transportation subsidies, and electronic reminders, regular follow-up phone calls, or text reminders could be used to further improve patient compliance.

## CONCLUSION

To sum up, in this first real-world study on the clinical effectiveness and safety of cinitapride among Chinese FD and FD-overlapping GERD, IBS, and FC population, our findings demonstrate that cinitapride is efficacious and well tolerated.
